# Supplementary material for: Ketosis Alters Transcriptional Adaptations of Subcutaneous White Adipose Tissue in Holstein Cows during the Transition Period
Source: Animals (Basel). 2022 Aug 30;12(17):2238. doi: 10.3390/ani12172238 (PMC9454750; doi:10.3390/ani12172238)
Supplement: Supplementary file 1 [file animals-12-02238-s001.zip › Supplementary Table S1.pdf]

Supplementary Table S1 GO enrichment analysis of DEG（FDR≤0.05）

Category	Term	Count	Gene	FDR
Ket PP vs AP up-regulated	inflammatory response	19	ENSBTAG00000014546, ENSBTAG00000008099, ENSBTAG00000027051, ENSBTAG00000019428, ENSBTAG00000012638, ENSBTAG00000015708, ENSBTAG00000008008, ENSBTAG00000008223, ENSBTAG00000009501, ENSBTAG00000020872, ENSBTAG00000020892, ENSBTAG00000019230, ENSBTAG00000002006, ENSBTAG00000022161, ENSBTAG00000004547, ENSBTAG00000003636, ENSBTAG00000019953, ENSBTAG00000014031, ENSBTAG00000019741	0.00367
Ket PP vs AP up-regulated	cytokine production	6	ENSBTAG00000013412, ENSBTAG00000027051, ENSBTAG00000032515, ENSBTAG00000003636, ENSBTAG00000037526, ENSBTAG00000021872	0.028177
Ket PP vs AP down-regulated	oxidation-reduction process	37	ENSBTAG00000008692, ENSBTAG00000016218, ENSBTAG00000012632, ENSBTAG00000009086, ENSBTAG00000005685, ENSBTAG00000005069, ENSBTAG00000020895, ENSBTAG00000000011, ENSBTAG00000006033, ENSBTAG00000009842, ENSBTAG00000001288, ENSBTAG00000020597, ENSBTAG00000004175, ENSBTAG00000026501, ENSBTAG00000026768, ENSBTAG00000007946, ENSBTAG00000015980,	2.47E-06

			ENSBTAG00000012072, ENSBTAG00000001727, ENSBTAG00000019603, ENSBTAG00000019025, ENSBTAG00000012012, ENSBTAG00000012232, ENSBTAG00000017779, ENSBTAG00000014127, ENSBTAG00000019419, ENSBTAG00000007754, ENSBTAG00000025540, ENSBTAG00000003279, ENSBTAG00000000042, ENSBTAG00000000448, ENSBTAG00000008747, ENSBTAG00000007812, ENSBTAG00000039582, ENSBTAG00000030335, ENSBTAG00000017253, ENSBTAG00000011990	
Ket PP vs AP down-regulated	hydrogen ion transmembrane transport	10	ENSBTAG00000008692, ENSBTAG00000043560, ENSBTAG00000043550, ENSBTAG00000043561, ENSBTAG00000012788, ENSBTAG00000014878, ENSBTAG00000000913, ENSBTAG00000009479, ENSBTAG00000039555, ENSBTAG00000002046	0.001721
Ket PP vs AP down-regulated	cholesterol biosynthetic process	8	ENSBTAG00000012059, ENSBTAG00000018936, ENSBTAG00000005069, ENSBTAG00000019512, ENSBTAG00000001592, ENSBTAG00000017819, ENSBTAG00000006589, ENSBTAG00000004075	0.010093
Ket PP vs AP down-regulated	mitochondrial electron transport, ubiquinol to cytochrome c	6	ENSBTAG00000043550, ENSBTAG00000019096, ENSBTAG00000022613, ENSBTAG00000000913, ENSBTAG00000009479, ENSBTAG00000012232	0.043662

Nket PP vs AP down-regulated	hydrogen ion transmembrane transport	9	ENSBTAG00000008692, ENSBTAG00000043560, ENSBTAG00000043550, ENSBTAG00000043561, ENSBTAG00000014878, ENSBTAG00000043556, ENSBTAG00000047768, ENSBTAG00000000913, ENSBTAG00000002046	0.00106
